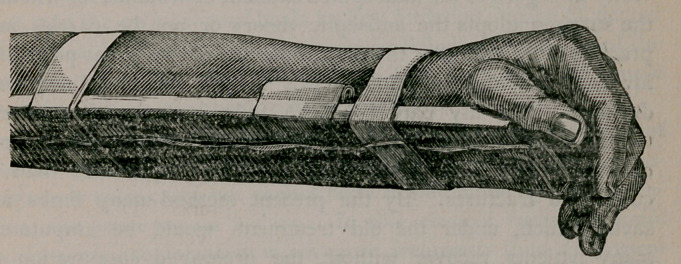# The Treatment of Fractures—An Exposition of the Most Recent Methods

**Published:** 1886-04

**Authors:** Hunter P. Cooper

**Affiliations:** Atlanta, Ga., Late Resident Assistant Surgeon to the Presbyterian Hospital, New York


### THE TREATMENT OF FRACTURES—AN EXPOSITION OF THE MOST RECENT METHODS.

By HUNTER P. COOPER, M. D., ATLANTA, GA.,

LATE RESIDENT ASSISTANT SURGEON TO THE PRESBYTERIAN HOSPITAL, NEW YORK.

The object of this paper is not to give expression to any originality in the treatment of fractures, but to describe somewhat in detail the methods pursued at the present day in hospital practice. To some it may appear presumptuous to discuss a subject which is already familiar. The large majority of general practitioners, however, do not treat many fractures, and know but little of the *technique* which modern surgery has perfected. When such physicians get a patient with a broken bone, their limited experience in this class of injuries forces them to review the subject in their text-books, and in consequence the patient gets the benefit of the methods of twenty years ago.

These remarks apply more particularly to compound than to simple fractures. I shall dismiss simple fractures after a consideration of only one, viz., Colles' fracture, and then proceed at once to the subject of compound fractures, for it is in the treatment of the latter that the greatest advances have been made.

THE TREATMENT OF COLLES' FRACTURE.

This variety of fracture has always been of great interest to surgeons, both because of its frequency as well as on account of the difficulty of treating it without resulting deformity or stiffness of the wrist joint.

Numerous splints have been devised and recommended in this fracture, many of which have their usefulness much impaired by their complicated structure.

Not being very familiar with the journal literature of the past few years, I am unable to tell who devised the splint I am about to describe. I know, however, that Dr. C. K. Briddon, of New York, was among the first to use it.

It is a splint which, while it fulfils every indication, is still of such simplicity that any one can fashion it with a pocket-knife and apply it in a few minutes.

It consists then of a single flat splint, made of bass-wood, poplar or soft pine. When applied it extends from just below the bend of the elbow to the middle of the metacarpal bones of the hand, along the anterior aspect of the forearm. The splint should be as wide as the widest part of the forearm, unless the patient is very muscular, in which case the sides of the splint converge slightly from above downward.

It is padded preferably with strips of old blanket or flannel. At a point on the splint corresponding with the lower end of the upper fragment of the radius, a compress of flannel is fixed by means of a strip of rubber plaster. The splint is now complete and ready to be applied.

Let us now see how to reduce the characteristic "silver-fork" deformity, for unless this be entirely overcome, our patient will always have an unsightly wrist, no matter what form of splint is used.

The directions given in Gross, Erichsen and Holmes for accomplishing reduction are exceedingly unsatisfactory when one attempts to follow them.

Gross says: "The fracture having been adjusted by pressure and extension," etc. Holmes is not much more explicit when he says: "The means by which . . . to remedy the displacement . . . consist in first reducing the fracture by traction on the hand and pressure on the fragment."

Erichsen says: "Forcible extension and counter-extension should be practiced with the view of disentangling the fragments and removing the dorsal prominence."

Each author then goes into a lengthy description of complicated splints, overlooking the fact that complete reduction is of far greater importance than any variety of splint. Of course, reduction is effected with more ease and with more marked benefit to the patient if it is done within a few hours after the occurrence of the injury. I am convinced that immediate reduction lessens the subsequent inflammation, and as a consequence less adhesion takes place between the tendons and their sheaths. In reducing the fracture, we should bear in mind the position that the fragments occupy and the nature of the obstacle to be overcome. The lower fragment is displaced backward, its lower end at the same time being tilted slightly upward. The posterior border of the upper fragment is more or less impacted into the cancellous structure of the lower fragment. Hence our first effort should be to disentangle the fragments. This is best done as follows: Grasp the patient's hand firmly, as if you would shake hands with him; the other hand is placed over the patient's wrist so that the thumb lies over the posterior aspect of the lower fragment, while the fingers lie over the anterior aspect of the upper fragment. While an assistant steadies the forearm, you carry the hand strongly backward—i. e., produce hyper-extension in order to disengage the upper fragment from the cancellous tissue of the lower. As soon as this is done, press the lower fragment forward with the thumb, counter-pressure being made by the fingers on the upper fragment. Simultaneously with this pressure, carry the hand rapidly into flexion, at the same time bearing towards the ulnar side, and the fracture is reduced. The ease with which reduction is accomplished by this method of hyper-extension will astonish any one who has struggled in vain to effect it by simple traction and pressure.

If then the deformity is entirely overcome, we are ready to apply the splint. This is fixed in position by two bands of rubber plaster one inch wide, one band being passed around the forearm and splint just below the elbow, the other band surrounding the lower end of the splint and passing directly over the posterior aspect of the lower fragment. The compress which has been fastened to the splint lies just at the lower end of the upper fragment, and by means of this compress and the lower band of plaster, pressure and counter-pressure is furnished to any degree needed, and a reproduction of the deformity is impossible. I should remark that no bandage is applied to the hand or forearm underneath the splint. A bandage, however, is applied *over* the splint from hand to elbow. The forearm is then put in a sling in a position of semi-pronation, the hand hanging free from the sling and not supported by it; this takes advantage of the weight of the hand to prevent deflection towards the radial side. If, however, this radial deflection cannot be entirely overcome, the lower end of the splint may be prolonged as the common pistol-splint, and the hand attached to this by plaster. The accompanying cut shows the splint applied to the forearm.

As a rule the splint can be removed on the 21st day; passive motion can be cautiously commenced at the end of two and a half weeks, and is to be done without removing the splint.

The advantages of this form of splint are:

1. Cheapness, simplicity of construction and ease of application—features which commend it highly to the general practitioner.

2. It obstructs the circulation very little; hence its use is attended with a minimum degree of swelling.

3. Sloughing of the skin from undue pressure is impossible.

4. It permits easily an examination of the parts by the surgeon without removal of the splint.

5. It allows some motion at the wrist and free motion of the fingers without disturbing the position of the fragments of bone.

6. It is impossible for this form of splint to slip out of place.

7. There results from its use very little false anchylosis of the wrist-joint and fingers, and this is soon overcome by passive motion.

After having seen this splint and others used in many cases of Colles' fracture, occurring in hospital and dispensary practice, I am convinced that the advantages stated above are real, not imaginary.

### THE TREATMENT OF COMPOUND FRACTURES.

It is in this class of fractures that the greatest advances have been made in recent years. Indeed, a complete revolution has been effected in their treatment since Lister promulgated his views in regard to the antiseptic treatment of wounds, for whether the surgeon adopts the antiseptic theory or not, he carries into practice more carefully than in former years two great principles involved in the application of this theory, viz., cleanliness and drainage. For my part I believe fully in antiseptic surgery, though I cannot go so far as the German extremists. In no class of wounds or injuries is antisepsis of more importance than in compound fractures. By the present method many limbs are saved which, under the old treatment, would be amputated; many patients recover without the prolonged suppuration so characteristic of the old plan.

There are practically two methods of dealing with a compound fracture antiseptically: 1, without wiring the fragments; 2, uniting the fragments by silver wire.

Let us suppose a case of compound fracture in which it is decided not to interfere with the fragments. If there is a chance of uniting the soft parts and converting it into a simple fracture, cleanse the limb about the seat of fracture with soap and water, and then wash it with a solution of bichloride of mercury (1-1500), pouring the same plentifully over the wound. If the soft parts are distended with clots, turn them out and irrigate the cavity with the bichloride solution, and trim the edges of the wound if much contused. The wound should then be closed with antiseptic catgut ligature, thickly covered with iodoform and a dressing of seven or eight layers of iodoform gauze applied. Over this a larger dressing of corrosive sublimate gauze is applied. Now the splint may be put on. The patient's temperature is to be taken night and morning, and this informs us accurately of the processes taking place underneath the dressing. So long as this is but little above normal, we may be sure that there is no suppuration or retention of septic products, and the splint should not be disturbed. A rise in temperature, however, above the slight traumatic fever, which is always present for one to three days after the injury, is the danger signal which nature hangs out to warn us that something is wrong at the seat of the wound. Then the dressing should be removed, and the cause will be readily discovered. As a rule, however, there will be no febrile rise if the antiseptic method has been carefully carried out, and the *entire* wound heals by first intention, or else its deeper parts heal in this way, leaving a superficial ulcer at the site of the wound. In either case the fracture has been rendered simple and the time of treatment greatly shortened.

In compound fractures, with great laceration or contusion of the soft parts, the antiseptic treatment possesses especial advantages; in such cases we cannot hope to get union by first intention, and hence we do not try. In this class of cases the knife was resorted to formerly much more frequently than at present in order to save the patient's life. If under the old plan it was decided to attempt to save the limb, the patient ran great danger of losing his life from osteo-myelitis and septicæmia, or, escaping these, from the exhaustion incident to prolonged suppuration.

By the antiseptic method osteo-myelitis and septicæmia are rarely encountered, and the stage of suppuration is markedly shortened. In detail it is pursued as follows: The hands of the surgeon having been thoroughly washed with soap and water, and then with a solution of mercuric bichloride or phenic acid, the finger is introduced into the wound and all loose splinters of bone removed. The patient's limb in the neighborhood of the wound is then cleansed as above described, and the wound itself thoroughly irrigated with a 1 to 1500 or 2000 solution of mercuric bichloride, the nozzle of the irrigator or syringe being introduced into the wound, so that the fluid will bathe it in every nook and cranny. It is best to have the antiseptic solution warm in order not to depress the vitality of the already contused tissues, and it should be used in great abundance.

Now inject by means of an ordinary syringe a mixture of glycerine and iodoform (four parts of glycerine to one part of iodoform). From ʒ ij to ℥ j of this should be injected, according to the size and depth of the wound; the excess is allowed to drain away, leaving, however, a thin layer of glycerine and iodoform over the wounded tissues, which forms a most efficient protection against decomposition of the discharges. If the wound is so situated that free drainage cannot take place, a counter-opening should be made at a dependent point, and a rubber drainage tube introduced and fixed in place.

If there is much hemorrhage, this is best controlled by stuffing the wound with iodoform gauze. The wound is then to be dressed with iodoform and corrosive sublimate gauze and the splint applied.

As regards the kinds of splints to be used in compound fractures, I think plaster of Paris, for cheapness and efficiency, is greatly to be preferred over other forms of splints in compound fractures of the lower extremity. In those involving the upper extremity, wooden, moulded leather, felt, gutta percha, or Levis metallic splints are more applicable.

A plaster of Paris splint can nearly always be applied to a broken limb at once, without waiting for inflammation to subside, but due allowance must always be made for the swelling which inevitably takes place. It should be done as follows: The whole limb is surrounded with a layer of cotton batting of uniform thickness, which is held in place by an ordinary muslin roller. Over this the plaster bandages may be applied without danger, for as the limb swells it compresses the cotton and thus makes room for itself. As a precaution the circulation in the toes should be closely watched, for this will give the earliest evidence of an impeded circulation in the limb. As soon as the swelling subsides, the splint should be removed, and another applied closely to the limb without the intervention of the cotton.

When we use plaster of Paris, a fenestrum is cut in the splint over the wound. In compound fractures of the leg and lower part of the thigh with extensive wound of the soft parts, a fenestrum sufficiently large to expose the whole of the wound would weaken the splint considerably; hence in such cases it is best to use Volkmann's bracketed plaster of Paris splint; this admirable splint allows the wound to be inspected and dressed without any inconvenience either to the surgeon or the patient.

The wound of a compound fracture should be dressed just as seldom as possible. The indication for a renewal of the dressing is given either by a rise of temperature or by the discharges soaking through the dressing.

Frequent renewals of the dressing delay the healing process; it is like digging up beans to see if they have sprouted.

The advantages of the antiseptic treatment of compound fractures may be summarized as follows:

1. The probability it affords of rendering the compound fracture simple.

2. The possibility of saving limbs, which otherwise would be lost.

3. It limits the inflammation (should any occur) within the bounds of healthy suppuration, thus saving the patient from the dangers of osteo-myelitis, cellulitis and septicæmia.

4. It shortens very materially the time of treatment.

The length of this paper precludes the possibility of introducing cases enough to prove these assertions, for it takes a great number of cases to demonstrate conclusively the efficacy of any form of treatment, but the whole of my experience with this class of injuries justifies me in believing in the correctness of my position.

There is still another method of dealing with compound fractures, viz., by drilling and wiring the fragments of bone together. Cases treated by this method have been reported in the journals from time to time, and from such reports my knowledge of the method has been derived. Drs. Wright and Hubbard, in the *New York Medical Journal*, of October 31, 1885, claim the following advantages for compound fractures of the bones of the leg and the patella treated by drilling the fragments and wiring them firmly together with silver wire:

1. The facility with which the wound may be cleansed of all matter likely to interfere with the healing without suppuration.

2. The possibility of effecting immediately perfect and permanent reduction of the fragments and the avoidance of irritation by them.

3. The avoidance of disturbing the parts by frequent dressings.

4. The greater probability of speedy union where the parts are securely immobilized.

5. The ability to save certain limbs, the seat of bad forms of compound fractures, which, under any other method of treatment, would demand amputation, or greatly endanger life by prolonged suppuration and its sequelæ.

In the same journal, January 16, 1886, Dr. Conway, of New York, reports two cases treated by wiring. The first was a case of compound fracture of the tibia, cured in twelve weeks. The second, a case of compound fracture of the humerus, with severe contusion and laceration of the soft parts, produced by the wheel of a street-car passing over the arm; perfect union without deformity was obtained in six weeks. In both cases the strictest antiseptic precautions were observed.

In conclusion I will add, that if any benefit is to be derived from the antiseptic treatment of compound fractures, it is only when it is carried out with scrupulous care and the closest attention to details.

## Figures and Tables

**Figure f1:**